# Elevated 4R‐tau in astrocytes from asymptomatic carriers of the *MAPT* 10+16 intronic mutation

**DOI:** 10.1111/jcmm.17136

**Published:** 2021-12-24

**Authors:** Núria Setó‐Salvia, Noemi Esteras, Rohan de Silva, Eduardo de Pablo‐Fernandez, Charles Arber, Christina E. Toomey, James M. Polke, Huw R. Morris, Jonathan D. Rohrer, Andrey Y. Abramov, Rickie Patani, Selina Wray, Thomas T. Warner

**Affiliations:** ^1^ Department of Clinical and Movement Neurosciences UCL Queen Square Institute of Neurology London UK; ^2^ Reta Lila Weston Institute UCL Queen Square Institute of Neurology London UK; ^3^ Queen Square Brain Bank for Neurological Disorders UCL Queen Square Institute of Neurology London UK; ^4^ Department of Neurodegenerative Disease UCL Queen Square Institute of Neurology London UK; ^5^ Neurogenetics Laboratory The National Hospital for Neurology and Neurosurgery London UK; ^6^ Dementia Research Centre Department of Neurodegenerative Disease UCL Queen Square Institute of Neurology London UK; ^7^ The Francis Crick Institute London UK

**Keywords:** 4R‐tau, astrocytes, frontotemporal dementia, frontotemporal lobar degeneration, pluripotent stem cells, tauopathies

## Abstract

The microtubule‐associated protein tau gene (*MAPT)* 10+16 intronic mutation causes frontotemporal lobar degeneration (FTLD) by increasing expression of four‐repeat (4R)‐tau isoforms. We investigated the potential role for astrocytes in the pathogenesis of FTLD by studying the expression of 4R‐tau. We derived astrocytes and neurons from induced pluripotent stem cells from two asymptomatic 10+16 carriers which, compared to controls, showed persistently increased 4R:3R‐tau transcript and protein ratios in both cell types. However, beyond 300 days culture, 10+16 neurons showed less marked increase of this 4R:3R‐tau transcript ratio compared to astrocytes. Interestingly, throughout maturation, both 10+16 carriers consistently displayed different 4R:3R‐tau transcript and protein ratios. These elevated levels of 4R‐tau in astrocytes implicate glial cells in the pathogenic process and also suggests a cell‐type‐specific regulation and may inform and help on treatment of pre‐clinical tauopathies.

## 1 INTRODUCTION

The microtubule‐associated protein tau gene (*MAPT*) exon 10+16 intronic mutation (IVS10+16C>T) causes autosomal dominant frontotemporal lobar degeneration (FTLD). Increased incorporation of *MAPT* exon 10 results in excess levels of the more fibrillogenic 4R‐tau isoforms in astrocytes and neurons.^1^ However, the underlying processes contributing to neurodegeneration are unclear. Glial cells, including astrocytes, play an important role in 4R tauopathy pathogenesis,^2^, ^3^ focussing on these cells in pre‐symptomatic cases could lead to identification of biomarkers for pre‐clinical detection of tauopathies. Induced pluripotent stem cells (iPSC) can be used to develop cell models to study underlying disease mechanisms and identify decisive factors in other frontotemporal dementia cases.^4^ In this study, we assessed long‐term expression of 4R‐tau mRNA and protein in iPSC‐derived astrocytes and neurons from asymptomatic carriers of the 10+16 *MAPT* mutation and affected post‐mortem brain.

## 2 MATERIAL AND METHODS

iPSC from two unrelated, asymptomatic female carriers with the 10+16 mutation (S1 and S2) and three healthy control cell lines were used as described previously.^5^ The iPSC were differentiated into neurons and astrocytes adapting previously established protocols^6^, ^7^, ^8^ and assessed at the same time‐points during maturation. Astrocyte‐specific function was demonstrated with calcium signalling following stimulation of astrocytic purinergic receptors and purity confirmed at different time‐points by immunocytochemistry (Figures S1 and S2). We also analysed post‐mortem brain tissue (Brodmann area 9) from an affected *MAPT* 10+16 mutation carrier and an age‐matched healthy control. For PCR analysis of *MAPT* exon 10 splicing, we used primers in flanking exons: Forward (exon 9): 5′‐GTCAAGTCCAAGATCGGCTC‐3′ and reverse (exon 13): 5′‐TGGTCTGTCTTGGCTTTGGC‐3, and amplification of GAPDH cDNA was used to normalise expression levels.^9^ Noting the presence of an additional band with agarose electrophoresis PCR products, we carried out fluorescent PCR with the *MAPT* forward primer (above), followed by denaturing capillary electrophoresis in a 3730XL (Applied Biosystems) and excluded the artefact (heteroduplex) for quantification (Figure S3). For protein analysis, cells were lysed using standard protocols. Lysates were dialysed into a 50 mM Tris‐HCl, pH 7.5 buffer and subsequently dephosphorylated using lambda protein phosphatase (NEB).^10^ Samples were analysed by Western blot with total tau antibody (Agilent Dako A002401‐2; 1:1000) and GAPDH antibody (Invitrogen; 1:10,000).

## 3 RESULTS

### 3.1 iPSC‐derived astrocytes 10+16 carriers show elevated mRNA 4R:3R‐tau ratio levels compared to controls

**FIGURE 1:**
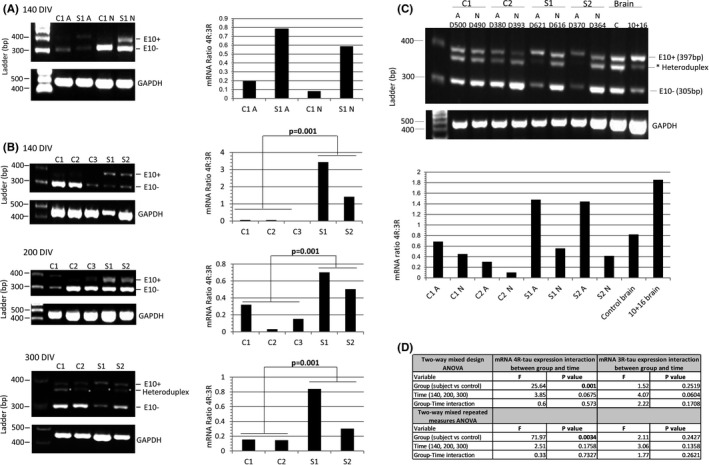
The mRNA 4R:3R‐tau levels in controls and 10+16 asymptomatic carriers at 140–300 DIV. (A) The mRNA 4R‐tau levels were analysed between astrocytes (A) and neurons (N) at 140 DIV. Ratios of 4R‐tau relative to 3R‐tau expression in astrocytes with or without mutation were higher compared with neurons at same time‐point. (B) Astrocytes at different DIV were analysed to determine the levels of 4R‐tau expression between controls and patients. Astrocytes with the 10+16 mutation expressed elevated 4R:3R‐tau mRNA ratio compared to controls and these values were consistent in the different time‐points. (C) Analysis of the 4R:3R‐tau mRNA between astrocytes and neurons after 370–620 DIV. Standard and fluorescent PCR of mRNA tau expression was performed to confirm the intermediate band (asterisk) as heteroduplex artefact and discard from quantification. The two PCR products at 397bp and 305bp correspond to 4R (exon 10^+^) and 3R‐tau (exon 10^−^), respectively. An affected 10+16 post‐mortem brain tissue and a healthy age‐matched donor samples were included in the analysis. The astrocytes (A) (with and without mutation) showed an increased 4R:3R‐tau mRNA ratio compared with neurons (N) at same time‐point. The 3R and 4R‐tau expression pattern differed also between controls and 10+16 carrier cell lines (also see Figure S3). (D) A Shapiro‐Wilk test was used to assess normality of distribution (Figure 1B). When variables were not normally distributed data were subjected to a log transformation. A two‐way mixed design ANOVA and a two‐way mixed repeated measures ANOVA was used to compare the results of the outcome variable (4R) with time (100, 200 and 300 DIV) as within‐patients variables and group (case versus control) as a between‐patients variable for three repetitions and four repetitions separately. The mRNA 4R‐tau expression in astrocytes at different time points showed statistically significant differences between carriers and controls and 4R‐tau expression did not show significant differences over time (140, 200 and 300 DIV) or the interaction between disease group and time. The mRNA results for expression in 3R‐tau showed no significant differences among cases vs. controls, over time or with the interaction between group and time (Figure 1B) (also see Table S1)

After 140 days in‐vitro (DIV) (Figure S4), both astrocytes and neurons derived from the asymptomatic *MAPT* exon 10+16 carrier (S1) showed elevated 4R‐tau mRNA (exon 10^+^) relative to 3R‐tau mRNA (exon 10^−^) compared to controls (Figure 1A). Semi‐quantitative analysis of *MAPT* mRNA at different time‐points showed significantly elevated 4R‐tau mRNA in astrocytes from both asymptomatic cases (S1 and S2) compared to controls (*p* = 0.001) and these differences persisted over 100, 200 and 300 DIV (*p* = 0.0034; Figure 1B,D). At much later time‐points (370–620 DIV), it is also striking that, though astrocytes maintained the increased 4R:3R‐tau mRNA ratio, in 10+16 neurons it decreased with time (Figure 1C). On agarose gels, we observed an intermediate band of about 380bp between those for the 3R and 4R‐tau mRNA bands (305 bp and 397bp, respectively). To resolve this, we used the fluorescent FAM‐labelled forward primer with capillary electrophoresis and demonstrated that this is a heteroduplex artefact, as previously described.^11^, ^12^ Of note is that during the course of the maturation of the astrocytes, the increased 4R:3R‐tau ratio in the 10+16 cases was less pronounced with the astrocytes from S2 than S1 (Figure 1B).

### 3.2 The 4R:3R‐tau protein isoform ratio is increased in astrocytes compared to neurons

**FIGURE 2:**
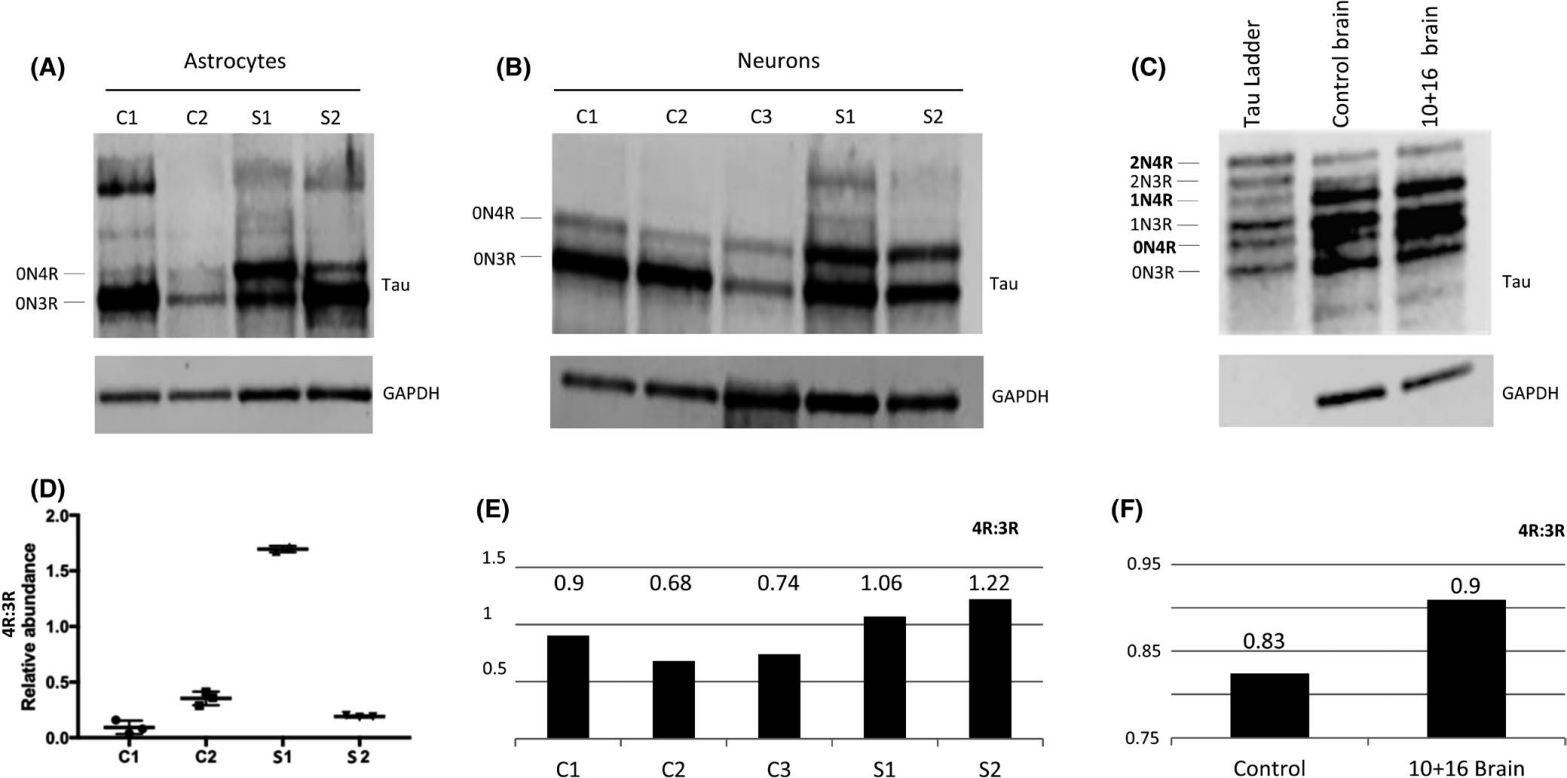
Protein analysis of total tau derived‐astrocytes and neurons at 300 DIV and brain donor samples. (A) Differences in tau isoform levels were identified in both astrocyte cell line carriers. The S1 sample showed increased 0N4R isoform compared to S2. (B) Increased 0N4R isoform was also seen in neurons, although S1 still showed an increased pattern of 0N4R compared to S2. (C) Frontal cortex samples from a 10+16 brain donor and control brain also showed increased 0N4R band in the 10+16 brain sample compared to control. (D) The statistical analysis used was ANOVA with post‐hoc Tukey. Significant differences in 4R‐tau levels between S1 and controls and S2 samples were identified (also see Figure S5). (E) Quantification graph of the western blot for total 4R:3R isoforms between controls and iPSC‐derived neurons after 300 DIV. The ratio values show increased 4R in both patients compared to controls. (F) Quantification graph of the western blot for total 4R:3R isoforms between healthy brain donor and 10+16 brain patient. The 10+16 brain patient show an increased accumulation of total 4R tau isoforms versus total 3R tau isoforms compared to healthy brain

The increased 4R‐tau in the 10+16 astrocytes is also seen at protein level at 300 DIV. Similar to mRNA levels, this is less pronounced with the astrocytes from S2 (Figure 2). In the control astrocytes, the major isoform is the embryonic 0N3R‐tau, with the 10+16 astrocytes showing increased 0N4R‐tau (Figure 2A,D). Interestingly, although the corresponding 10+16 neurons at the same time‐point show increased 0N4R‐tau compared to controls (Figure 2B,E), this is to a lesser extent than in the astrocytes. As with the astrocytes, the ratio of increased 4R:3R‐tau is lower in the neurons from the S2 asymptomatic 10+16 case compared to S1. Western blot of cortical tissue from post‐mortem brain of a 10+16 FTLD case also demonstrated increased 4R‐tau (Figure 2C,F).

## 4 DISCUSSION

This study of iPSC derived astrocytes and neurons from two 10+16 mutation carriers identifies astrocytic phenotypes that may help understand disease pathogenesis in FTLD, with aberrant expression and splicing of 4R tau. In this study, we have shown that astrocytes derived from iPSCs from two carriers of the 10+16 mutation have increased 4R‐tau, which is known to lead to tauopathy. We demonstrated *MAPT* expression in both 10+16 and control astrocyte cultures, with detectable and increasing 4R‐tau mRNA during maturation in 10+16 cell lines. The increased 4R‐tau production in 10+16 astrocytes persisted at all the time‐points of our analysis. As we have previously reported, neurons also had increased 4R:3R‐tau mRNA ratio compared to controls,^5^ but with time, the ratio decreased, whilst in astrocytes, the increased ratio was maintained. This may reflect an autonomous astrocytic role in the evolving 4R‐tau pathogenesis of 10+16 FTLD, rather than passive uptake of abnormal tau protein from neurons. Nevertheless, several questions remain unresolved and other factors may be involved, including differences in genetic and/or epigenetic factors as well as environmental influences driving post‐transcriptional processes, and the interaction between neurons and glia in vivo.^13^, ^14^

We also demonstrated increased 4R‐tau protein in the 10+16 astrocytes and neurons compared to controls, which was mirrored in frontal cortex from a brain with 10+16 FTLD. It is of interest that one of the 10+16 carriers (S2) consistently showed less pronounced increases in 4R‐tau mRNA and protein compared to the other carrier (S1). This variable expression of the mutant allele could be due to differences in genetic background between the two carriers. For example, S1 is homozygous for the *MAPT* H1/H1 haplotype that is the strongest genetic risk factor for PSP and CBD whereas, S2 is heterozygous, H1/H2.^5^ It is plausible that the protective H2 haplotype exerts an epistatic effect on its opposing mutant allele. Therefore, our findings raise the possibility that underlying genetic factors play a role in influencing the functional effect of the 10+16 mutation on *MAPT* exon 10 splicing and affect expressivity and cellular and clinicopathological phenotype, as described in other tauopathies.^3^, ^15^

Further studies to identify mechanisms specific to astrocytes are required to confirm these results and better understand the sequence of cell‐specific events.

## CONFLICT OF INTEREST

The authors declare no competing interests.

## AUTHOR CONTRIBUTION

**Nuria Seto‐Salvia:** Data curation (lead); Formal analysis (lead); Investigation (lead); Methodology (lead); Validation (lead); Visualization (lead); Writing – original draft (lead). **Noemi Esteras:** Data curation (supporting); Formal analysis (supporting); Methodology (supporting); Validation (supporting); Writing – original draft (supporting). **Rohan de Silva:** Formal analysis (supporting); Methodology (supporting); Supervision (supporting); Writing – review & editing (equal). **Eduardo de Pablo‐Fernandez:** Data curation (supporting); Formal analysis (supporting); Writing – original draft (supporting). **Charles Arber:** Data curation (supporting); Formal analysis (supporting); Funding acquisition (supporting). **Christina E Toomey:** Data curation (supporting); Methodology (supporting). **James M Polke:** Formal analysis (supporting); Methodology (supporting); Software (lead); Validation (supporting). **Huw R Morris:** Writing – original draft (supporting). **Jonathan D Rohrer:** Writing – original draft (supporting). **Andrey Abramov:** Writing – review & editing (supporting). **Rickie Patani:** Conceptualization (supporting); Supervision (supporting); Writing – review & editing (equal). **Selina Wray:** Conceptualization (lead); Funding acquisition (equal); Resources (equal); Supervision (supporting); Writing – review & editing (equal). **Thomas T Warner:** Conceptualization (lead); Funding acquisition (lead); Resources (lead); Supervision (lead); Writing – review & editing (lead).

## Supporting information

Fig S1‐S5 and Table S1

## Data Availability

The data that supports the findings of this study are available in the supplementary material of this article.
