# Supplementary material for: Elevated 4R‐tau in astrocytes from asymptomatic carriers of the MAPT 10+16 intronic mutation
Source: J Cell Mol Med. 2021 Dec 24;26(4):1327–31. doi: 10.1111/jcmm.17136 (PMC8831975; doi:10.1111/jcmm.17136)
Supplement: Supplementary file 1 — Fig S1‐S5 and Table S1 [file JCMM-26-1327-s001.docx]

**Supporting Figure 1**


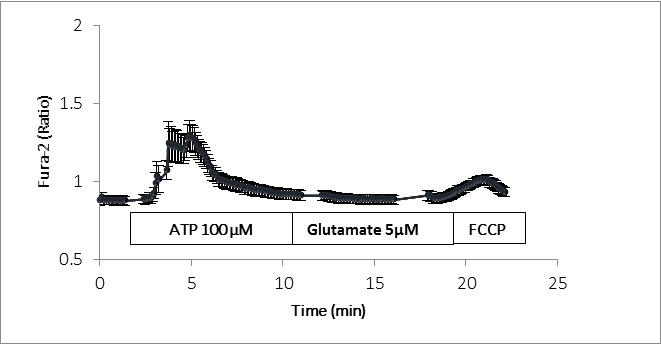

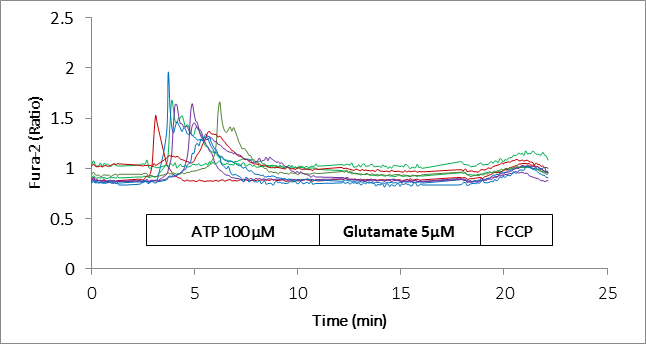


A

B

**Supporting Figure 1.** Fluorescent measurements of Fura-2 in astrocytes were obtained on an epifluorescence inverted microscope coupled to a CCD camera and [Ca^2+^] was monitored in single cells using excitation light provided by a xenon arc lamp and the beam passing monochromator at 340 and 380 nm (Cairn Research, Kent, UK). The number of cells showing an increase in the ratio 340/380 nm were counted and divided by the total number of astrocytes. All cell lines were tested using this method. Typically 50-200 cells were analysed per experiment in a total of 17 experiments in control cells and 21 in mutants. ATP induced a calcium response in 45±7% control and 54±7% 10+16 subjects’ iPSC-derived astrocytes. The Shapiro-Wilk test confirmed normal distribution and one-way ANOVA with Bonferroni post-hoc was performed in OriginPro 2019 software. No statistically significant differences were found between them (controls, mutants and primary astrocytes (ScienCell) with p=0.98, p=1 and p=1, respectively), and the percentage of responding cells were also similar to those obtained when analysing the ATP-induced calcium response of commercial human primary astrocytes (33±9%, n=2). **A**: Representative graph of the average number of cells (control) used in the experiment. **B**: Representative graph of individual cells (control) used in the experiment.

**Supporting Figure 2**


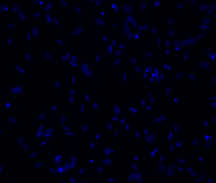

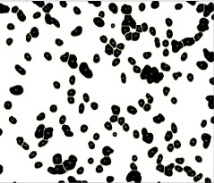

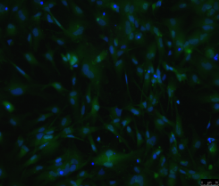


C

DAPI

EAAT1/DAPI

A

C1

DAPI

EAAT1/DAPI


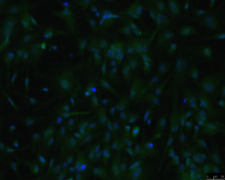

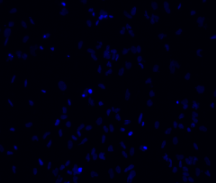

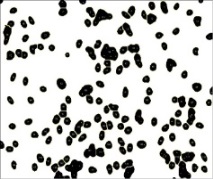


S1

Number of cells

Image 1 Image 2 Image 3 Image 4 Image 5


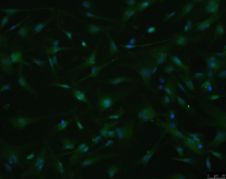

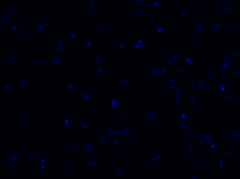

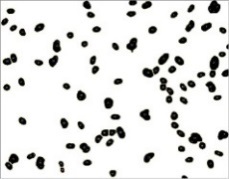


DAPI

EAAT1/DAPI

S2

B


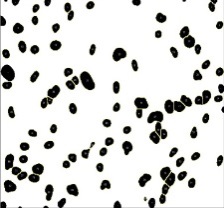

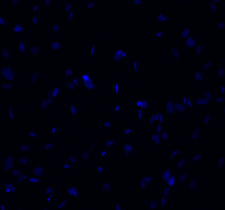

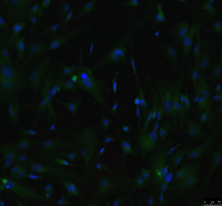


GFAP/DAPI

DAPI

C1


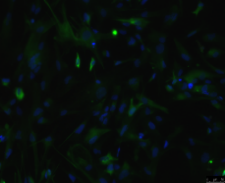


DAPI

GFAP/DAPI


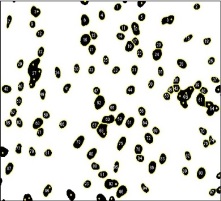

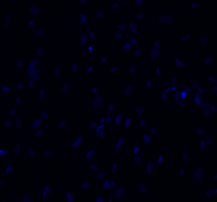


Number of cells

S1


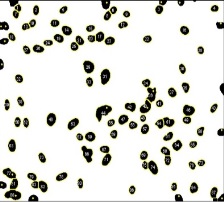

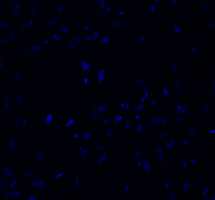

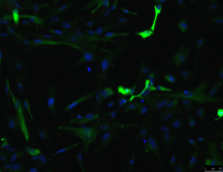


DAPI

GFAP/DAPI

Image 1 Image 2 Image 3 Image 4 Image 5

S2

**Supporting Figure 2.** Representative images of the astrocytes labelled with specific antibody (**A**: EAAT1 1:1,000, and **B**: GFAP 1:1,000). Image J software was used for cell recognition with specific threshold for cellular shape and for the counting cells positive for each specific astrocyte marker. **C**: The graphic of all positive staining iPSC-derived astrocyte cells using both antibodies.

**Supporting Figure 3**


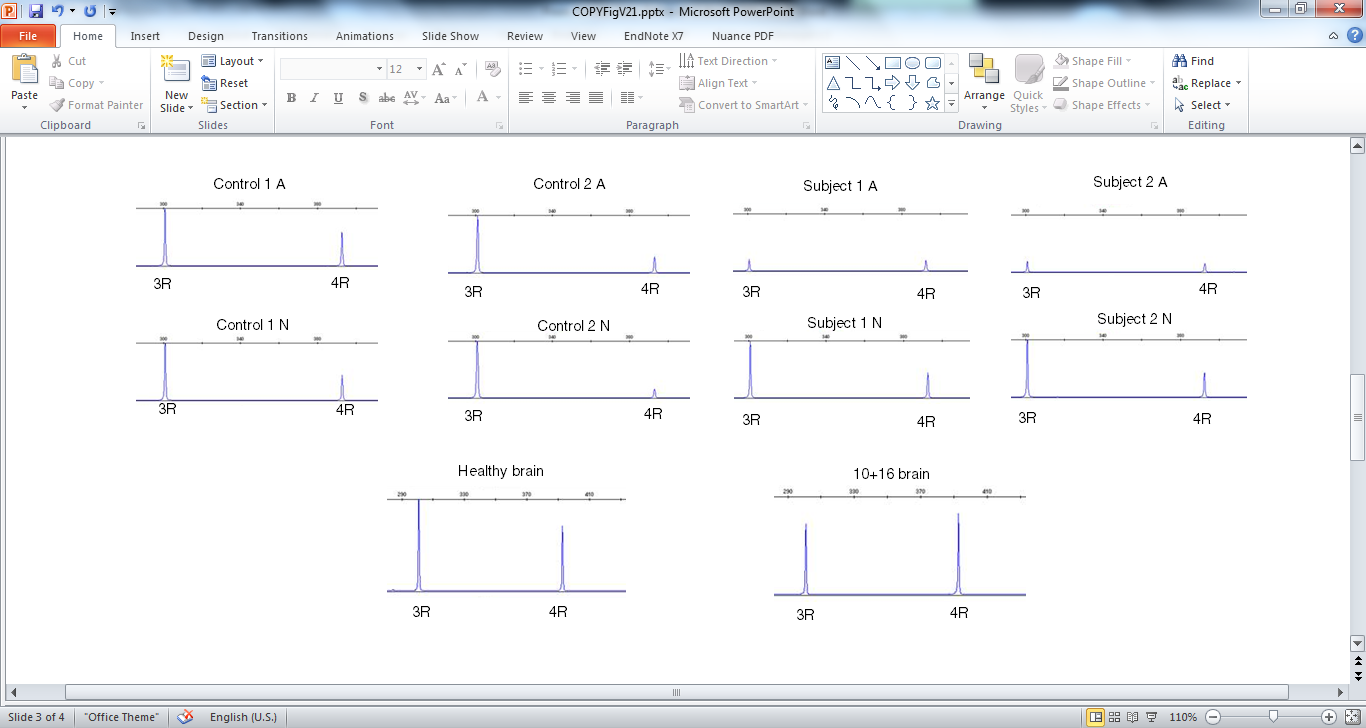


**Supporting Figure 3.** We analyzed the astrocyte and neuron after 370-620 days in vitro, using the fluorescent FAM-labelled forward primer. Two bands were detected at 397bp and 305bp corresponding to 4R and 3R-tau, respectively. The results show absence of intermediate band, and confirming the presence of an artefact (heteroduplex) in the agarose gel, which were excluded in all analyses.

**Supporting Figure 4**


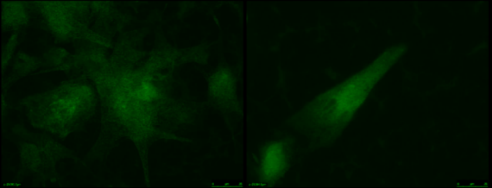

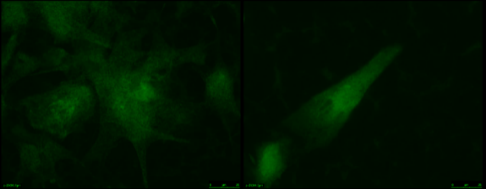

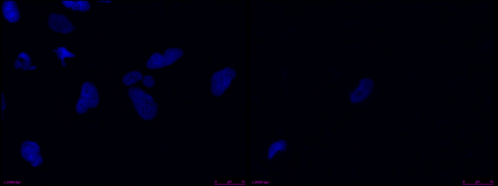

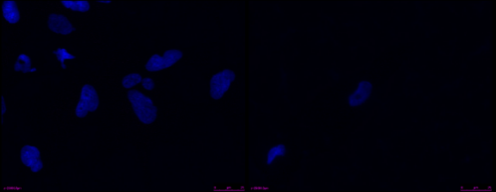

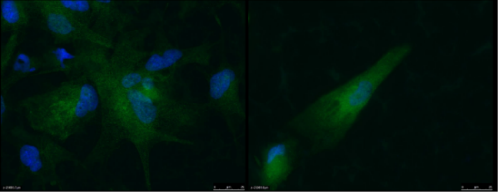

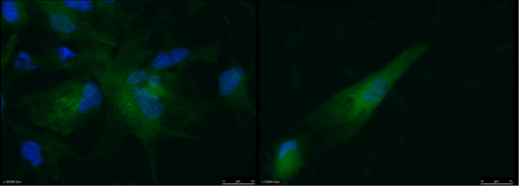


T. TAU

DAPI

T. TAU / DAPI

T. TAU

DAPI

Control

Subject


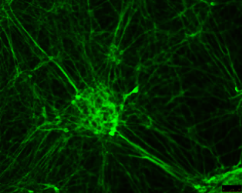

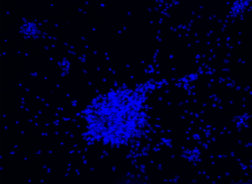

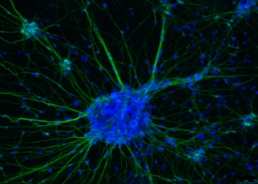

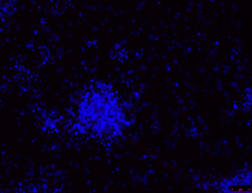

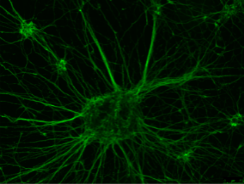

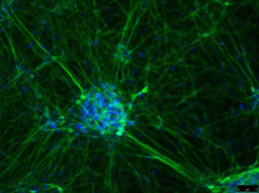


T. TAU / DAPI

T. TAU

DAPI

T. TAU / DAPI

T. TAU

DAPI

Control

Subject

A

T. TAU / DAPI

B


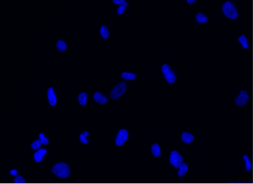

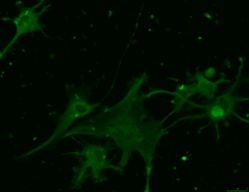

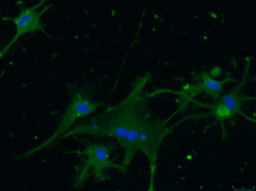

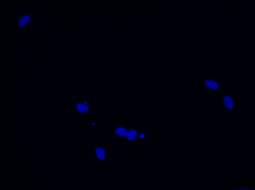

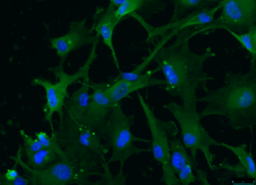

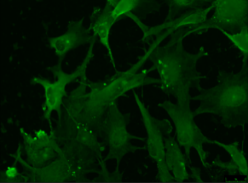


T. TAU / DAPI

DAPI

T. TAU

Control

Subject

C

60x

T. TAU / DAPI

DAPI

T. TAU

**Supporting Figure 4.** The immunocytochemistry of total tau antibody (Agilent Dako A002401-2; 1:1,000) was used in astrocytes (**A**) and neurons (**B**) at 140 DIV for the identification of endogenous tau. DAPI was used for nuclear staining in all samples. **C**: Astrocyte cell lines were stained after 300 days in culture using same antibodies. No differences in morphology or tau expression were found between controls and 10+16 carriers at this stage.

**Supporting Figure 5**

**
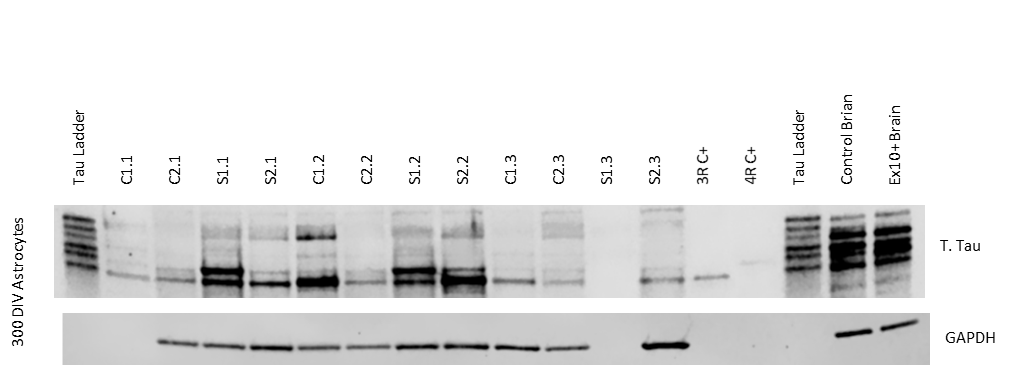
**

**Supporting Figure 5.** Western blot of tau protein in astrocytes analysed at 300 DIV (**A**). The quantification of the protein bands was performed using the software Prism8 (GraphPad) and the statistical analysis used was ANOVA with post-hoc Tukey (also see Supporting Table 1).

**Supporting Table 1**

**
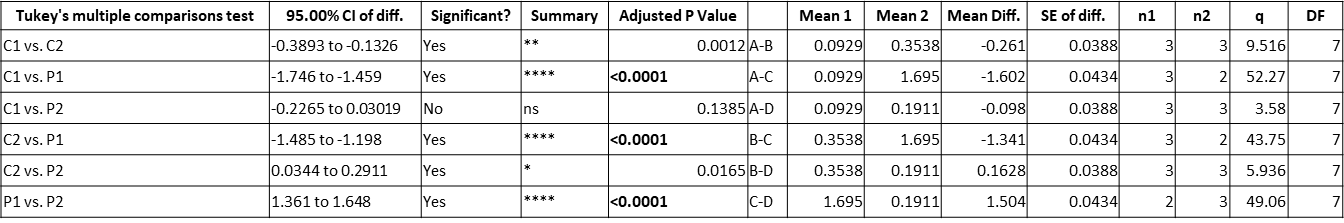
**

**Supporting Table 1.** The statistical analysis used in supporting Figure 5 was ANOVA with post-hoc Tukey. Significant differences in 4R-tau levels between S1 and controls and S2 samples were identified (also see Figure 2D).
